# A Qualitative Investigation on the Roles of Social Support on Physical Activity Behaviour among the Rural-Dwelling Older Women in Malaysia

**DOI:** 10.3390/ijerph18189609

**Published:** 2021-09-12

**Authors:** Thaneswaran Marthammuthu, Farizah Mohd Hairi, Wan Yuen Choo, Nur Afiqah Mohd Salleh, Noran Naqiah Hairi

**Affiliations:** 1 Centre for Epidemiology and Evidence Based Practice, Department of Social and Preventive Medicine, Faculty of Medicine, University of Malaya, Kuala Lumpur 50603, Malaysia; ccwy@um.edu.my (W.Y.C.); imohdsalleh@um.edu.my (N.A.M.S.); noran@um.edu.my (N.N.H.); 2 Ministry of Health, Putrajaya 62590, Malaysia; 3 Faculty of Public Health, Universitas Airlangga, Mulyorejo, Surabaya City 60115, Indonesia

**Keywords:** older women, physical activity, qualitative, rural, social support

## Abstract

Despite many health benefits of physical activities, both physically and mentally, the majority of the older women in the rural areas of Malaysia are showing a low prevalence of physical activities. Understanding the roles of social support to improve physical activities is imperative to promote active and healthy ageing among the rural-dwelling older women in Malaysia. Hence, this qualitative study adopted an inductive design using 17 in-depth interviews to understand the role of social support on physical activity behaviour among the rural community-dwelling older woman in Kuala Pilah District, Negeri Sembilan, Malaysia from December 2019 to January 2020. Three categories of themes were identified in this study. Firstly, adaptive social support in terms of informational, companionship and emotional supports reported enhancing physical activity levels among older women. Secondly, the absence of help and assistance from the social network asserts independence and triggers the older women to perform the activities by themselves, thus engage in physically active lifestyles. Thirdly, lacking social support demotivate older women to be engaged in physical activities. In particular, loss of companionship support, poor acceptance or appraisal support, logistic issues to attend exercise programmes and neighbourhood safety and security issues were among the main barriers of physical activities reported by the older women. The main findings of this study shed some light on the exigency of strengthening social support from the social network to engage the older women in physical activities. The roles of social media, effective patient-doctor communication and interventions targeting the spouses and family members must be strengthened to create a supportive atmosphere to enhance physical activity levels among older women.

## 1. Introduction

In line with the contemporary global statistics, most countries in the world were projected to experience an increase in the proportion of older people, from 1 in 8 people aged 60 years or above in 2017 to 1 in 5 by 2050 [1,2]. In specific, gender differences in life expectancy suggest that women will make up a large proportion of the world’s older population due to differential genetic vulnerability to illness, reproductive and hormonal factors as well as differences in physiological, psychological and sociological characteristics during their life-cycle [3,4,5]. With life expectancy of the women projected to reach 83.0 years by 2040 [3], a larger proportion of older women will be occupying most of the continents in years to come compared to their counterparts. A similar trend is observed in Malaysia, an upper-middle-income country where the number of older females above 60 years old dominate the old age group by 2.2 million compared to the males [6]. It can be elucidated that older women’s vulnerability to poor health in older age is high and therefore, it is crucial to opt for potential options that promote active and healthy ageing among them. Physical activity of older women is envisaged as a key for active ageing while improving cognitive and physical functioning [7,8,9]. According to WHO guidelines, adults aged 65 years and above are recommended to do at least 150 min of moderate-intensity aerobic physical activity throughout the week or engage in at least 75 min of vigorous-intensity aerobic physical activity throughout the week or an equivalent combination of moderate- and vigorous intensity activity [9]. A review of physical activity from 168 countries found that 27.5% of the sample were physically inactive [10]. In addition, physical activity prevalence among the older women is of low intensity and tend to confine housework and less vigorous activities compared to their male counterparts [11].

A plethora of studies has investigated the multitude of factors that induce older adults to initiate and maintain physical activity. These factors are often divided into those that are invariable (sociodemographic characteristics) and those that are presumed to be modifiable (behavioural and personality attributes, environmental factors and community settings) [12,13]. A number of studies that focused on older women have demonstrated that built environment aspects [14,15], major life events [16], older women’s beliefs about physical activity [17], husbands’ influences [18] and neighbourhood walkability [19] to influence the physical activity behaviour among them. The health status of the older women and existing medical implications also reported shaping their physical activity behaviour [20,21,22]. Therefore, identifying factors that influence older women’s physical activity behaviour is crucial to promote active, healthy or successful ageing [23]. In line with this, social support, which is a social determinant of health, identified as an important modifiable factor influencing physical activity levels among older adults [24,25].

Social support forms the basis of the reciprocal social relationships between the older adults and the members of their social network to encourage them to participate in physical activities. The concept of social support is grounded on four multifaceted domains including emotional, informational, companionship and tangible support [26]. Emotional support in terms of love, affection, empathy, concern, caring, trust, acceptance, intimacy and encouragement by the social network provides the older adults with a sense of being valued or appreciated by the social members [27]. Having supportive family members and friends were found to be associated with increased inclination towards physical activity among the older women [27]. Informational support refers to the provision of advice, guidance, suggestions as well as valuable information to the older adults in order to motivate them to encourage them to involve in physical activity according to the recommendations [28]. Companionship support provides the older adults a sense of social belonging, social inclusion or integration and motivate them to engage in group activities such as leisure, exercises and sports [29]. Tangible support refers to the acquisition of resources in the form of financial assistance, materials and services from the social network to support the older adults to be involved in physical activities [30]. Essentially, the attainment of healthy ageing entails proper physical activity and strong social support, measured by these domains. [31,32,33]. For instance, regular physical activity and higher levels of social support were associated with reduced psychological distress findings among the community-dwelling older adults in Ghana [33]. Another study performed in South Korea reported similar significant findings of a positive association between perceived social support and physical activity among active older adults [34]. However, a randomized controlled intervention in Canada demonstrated no significant relationship between social support from family and physical activity among older adults. The authors described the non-significant association was due to the influence of small sample size and variations in the degrees of social support and physical activity among the study participants. In a qualitative assessment of examining Canadian Punjabi Sikh men’s experiences of adopting lifestyle changes following myocardial infarction (MI), the family support is found to correlate with a changed behaviour associated with physical exercise [35]. In a review of 27 articles, Smith [25] reported a positive association between social support and physical activity levels in older adults, especially when it comes from family members. In addition, leisure time physical activity was associated with social support in a greater number of studies compared to other physical activity domains.

Even though the relationship between social support and physical activity is well reported in many quantitative studies, the influence that social supports exert towards shaping the physical activity behaviour among older adults warrants more evidence in the rural and urban contexts. Furthermore, qualitative studies that assess the opinions, thoughts and feelings of the older adults towards the social support received for the physical activity is limited to the realm of gerontology studies and needs to be investigated in different regional contexts, especially in the middle- and low-income countries. Since older women are one of the vulnerable populations that need to be targeted to promote active ageing, the present study aimed to explore the roles of social support on physical activity behaviour among the rural community-dwelling older woman in Kuala Pilah District, Negeri Sembilan, Malaysia. The findings of this study envisaged providing impactful insights and information for the healthcare planning to devise necessary actions and educational campaigns to accelerate adequate social support as one of the strategies to boost physical activity levels among the older women.

## 2. Materials and Methods

### 2.1. Study Design and Settings

An inductive qualitative design using semi-structured interviews was adopted in this study to gain comprehensive insights on the roles of social support towards the physical activity behaviour among the rural community-dwelling older women in Kuala Pilah District, Negeri Sembilan. The study was conducted in the traditional villages and residential areas located in the outskirts of Kuala Pilah, an old valley town surrounded by traditional style Malay village houses built on stilts nestled among extensive rice fields in Malaysia. The data collection was carried out from December 2019 to January 2020 in the houses of the respective older women who have registered their willingness to participate in this study.

### 2.2. Study Sample

This study utilized a combination of purposive and snowball sampling approaches in the sample recruitment. The study samples recruited in the current study was a subset of the participants who were involved in a rural community-dwelling older persons’ (age 60 and older) cohort study in Malaysia (MAESTRO project) from 2013–2020. Potential older women who have participated in the Malaysian Elder Mistreatment Project (MAESTRO) cohort study were identified, screened for the inclusion and exclusion criteria and re-invited via phone calls to participate in the present study. The inclusion criteria set in the study were those aged 60 years and above, residing in the community for more than 12 months as well as able to converse in the local dialects or languages (e.g., Malay, English or Tamil). The exclusion criteria were older women residing in care facilities and those with impaired and borderline cognitive function assessed with Mini-mental State Examination (MMSE) scores less than 24.

### 2.3. Data Collection

A semi-structured interview guide corresponding to the research questions as illustrated in Table 1 was used to assist the moderator to direct the flow of the interviews. Generally, the interview guide comprised of probes that examine participants’ perceptions and ideas on the types and importance of support received from their social network to be engaged in physical activities. The guide was reviewed by three independent researchers and pilot tested on randomly selected older women who were not part of the present study. The participants were well-informed of the study objectives and that their participation was on a completely voluntary basis. The confidentiality, anonymity, privacy and respect to the participants were prioritized and reassured throughout the study. In addition, informed consent was obtained from all the participants before the interview sessions. All the data collected from the interviews were carefully stored in a password-protected database that can be only accessible by the researchers. The interviews were held at the participants’ homes with respect to their mobility and logistic options. The interviews were conducted by the first author. Essentially, the interviews were conducted in the spoken language that the participants were most comfortable with. The participants were also encouraged to express their inputs narratively to ensure the retrieval of all the necessary information associated with the study objectives. The majority of the interviews were conducted in Malay, followed by Tamil and English languages before cautiously translated into the English language for data analyses. A series of interviews were continued until data saturation was achieved and no new information was elicited. All interviews were audiotaped and transcribed verbatim. Additional notes, including the participants’ facial expressions and kinesics, were collected by the note-takers (research assistants who are trained in qualitative data collection process). The first author transcribed all the audiotaped interviews. The transcripts were checked for accuracy and completeness by listening to the tapes and comparing them to the transcripts by the other authors. A summary of the key points obtained during the interviews was presented to the participants for clarification. Upon completion of each interview, the participants were rewarded with one kilo of organic rice and a hand wash as tokens of appreciation.

### 2.4. Data Management and Analysis

All the interviews were conducted separately, transcribed and analysed to determine saturation point [36]. All the audio recordings which were not in English were transcribed and translated into English by the authors before analysed further. All interviews were compared with the analysis of previous interviews which in turn further shaped the subsequent sampling, data collection and analysis until no new information was uncovered [37]. Thematic saturation was achieved after 15 interviews were completed and two additional interviews were added to ensure that adding more interviews will not elicit any explicit information on the role of social support towards the physical activity behaviour of rural community-dwelling older women [38,39]. Coding and categorizing were carried out using NVivo version 11.0 in this study. The principles of content analysis were applied where transcripts were read several times and coded [40]. Similar codes were grouped and re-grouped as the study progressed. Groups and sub-groups were named as themes and sub-themes according to the key findings [40]. The themes and sub-themes were discussed by the authors to ensure that the older women’s worlds were fully captured. The first author performed the initial data analysis and the other authors coded independently and the themes and sub-themes were compared [40]. Discrepancies in coding and theme development were discussed and a consensus was reached that best suited the data generated. Lastly, the results of the 17 interviews were analysed, interpreted and presented using the participants’ own words as illustrations.

### 2.5. Ethical Consideration

The ethical clearance was obtained from University Malaya Research Ethics Committee (UM.TNC2/UMREC_1189). Prior to the study commencement, the study instruments and protocols were carefully reviewed and approved by the Medical Research Ethics Committee (MREC), Ministry of Health, Malaysia and registered under the Malaysian National Medical Research Register (NMRR-20-2769-57605).

## 3. Results

### 3.1. Background of the Participants

A total of 17 interviews were conducted from December 2019 to January 2020 to examine the role of social support in influencing the physical activity behaviour among the rural community-dwelling older women in Kuala Pilah District, Negeri Sembilan. The detailed description of the socio-demographic attributes of the study participants was depicted in Table 2. The mean age of the participants was 74 ± 5.9 years and ranged from 60 to 90 years. In addition, the majority of participants were Malays (*n* = 13, 76.5%) and Muslim (*n* = 13, 76.5%). More than half (*n* = 12, 71%) of the study participants completed their secondary education and all of them were married. A large proportion of the participants (*n* = 14, 82.4%) included in this study were housewives.

### 3.2. Roles of Social Support on Physical Activity Behaviour among the Rural Community-Dwelling Older Women

Five major themes were identified such as informational support to the older women to do physical activities, companionship support to engage with shared physical activities, emotional support from the surrounding people, the absence of social support as a motivator to engage with physical activities as well as lacking social support and less supporting social environment as barriers of physical activities among older women. These themes were grouped into three main categories such as adaptive social support that encourages physical activity (three themes), poor social support that encourages physical activity (one theme) and poor social support that discourages physical activity (one theme) among the rural community-dwelling older women.

#### 3.2.1. Adaptive Social Support That Encourages Physical Activity among Rural Community-Dwelling Older Women

##### Informational Support to the Older Women to Do Physical Activities

In general, participants reported informational support such as advice, guidance, suggestions and other useful information concerning physical activities as a form of social support. Informational support received from friends, media and health practitioners offered knowledge for participants to be engaged with the physical activity. They registered that information on the types and benefits of physical activities have allowed them to become more informed, therefore motivating them to create changes in physical activity levels. In relation to this, some participants expounded that they were advised by their friends to engage with group physical activities as a form of exercise to stay healthy. The participants highlighted that they were occasionally reminded by their friends that being physically inactive could elevate the possibility of developing functional dependence on other family members. One of the participants expressed this as:

“My friend always advises me that rather than staying at home and depending on others, we can go shopping and buy things ourselves. This can make our body healthier”

Indian, 76-year old

“… she advises that walking in the morning is good for a diabetic patient like me to control my sugar levels and to perspire”

Malay, 68-year old

On another occasion, some participants registered that they will be often persuading their friends and sharing their experience to indulge in regular exercises during their leisure time to stay active and healthy. For instance, one of the participants conveyed this as:

“I always like to invite my friend to join the exercise. I’m not a professional but, I would like to advise and guide her on the benefits of exercise based on my experience”

Chinese, 73-year old

The findings also suggested that media interventions and awareness programmes broadcasted in television health shows, campaigns and news targeting older people as an efficient mass-reaching communication tool to impart them adequate information on the benefits of physical activities. In particular, religious programmes that stress the importance of active lifestyles broadcasted on the television and radio channels were reported as one of the main motivators for older women to engage in physical activities. Specifically, they recollected their views that being physically active was regarded as one of the Sunnah of the Prophet and adherence to the Sunnah was indispensable for religious, social and health benefits.

“Sometimes, the Ustaz (religious teacher) in the television programmes asks us to exercise for at least 30 min in a day. On top of that, exercises are regarded as Sunnah from Nabi”

Malay, 69-years old

Being a primary mode of entertainment for older women in rural areas, television programmes play a role as the dominant source of information and motivation to engage in physical activities. For example, some of the participants registered that the media occasionally relayed information of the health risks due to inactivity such as obesity, diabetes and hypertension that strike most of the Malaysians. Additionally, they propounded that television programmes are also key in creating awareness of the necessity of keeping an active lifestyle at an older age. In addition to the television, some participants registered the efficiency of Facebook and YouTube channels in providing good exposure for them on what kinds of exercises and physical activities suitable for the older people.

“We follow Facebook and YouTube channels to check on what kind of exercise we can do; we do what we can do”

Chinese, 73-year old

The information and suggestions on the types of physical activities for older people by the health care professionals were outlined as another primary support for the older women to engage with physical activities.

“The doctor suggests me to go for a jog every day. I replied that I can’t run at this age. She advised me that I could walk slowly. I can sweat if I walked for about 30 min at least”

Malay, 69-year old

The participants described that healthcare professionals often reminded them to keep physical activity as a routine activity to remove non-communicable diseases at the older age. Some participants described that their doctors will be asking questions to monitor their physical activity levels during regular medical check-ups to support them in becoming back on track if they experience lulls in physical activity.

“The doctor suggests me to do simple housework to stay active at home. He discourages me to sleep a lot, but will remind to take sufficient rest”

Malay, 68-year old

##### Companionship Support to the Older Women to Engage with Shared Physical Activities

The companionship of peers has been reported to encourage engagement in physical activities among study participants. For example, friends will invite them to participate in joined activities such as evening walks, buying groceries from nearby sundry shops as well as walking to the temples. Some participants stated that walking with friends who often request them to walk enable them to be engaged with physical activities. They perceived that companionship provided them with a sense of social belonging while undertaking physical activities with their peers. Eventually, this enhances the social functioning that reduces greater levels of societal isolation, loneliness and depression that they encounter at their older age.

“In the mornings, my friends invite me to join them for a walk. They usually reprimand me not to sit and focus on my sewing work. They will ask me to join them and to be physically active”

Malay, 68-year old

“My friends often invite me to join aerobic exercises for the older people in Klinik Kesihatan Kuala Pilah (Kuala Pilah Clinic). They usually remind me that I might feel bored and lonely if stay home. I also realized that I am not alone by joining their activities (grinning)”

Malay, 70-year old

##### Emotional Support from the Social Network to Engage the Older Women with Shared Physical Activities

The findings implied that the availability of emotional support from the social network comprised of family members and healthcare practitioners play a crucial role in determining the active lifestyle among older women. For instance, some of the participants elucidated that they feel being loved and cared for when the family members support and help them to perform activities that they love to do such as cooking, gardening and sewing. They also indicated that they feel being appreciated and valued when the family members were taking care of their health and well-being. In addition, the love and happiness provided by the families, friends and neighbours trigger them to be actively engaged with the small activities that they prefer to perform in their daily routine.

“My daughter always advise me that I need to take care of my health as she is concerned about my well-being. She will encourage and assist me to prepare food in the kitchen during weekends. My family members always say that they love to eat my cooking (smiling). I was very touched by their words and this motivates me to prepare meals for them every day”

Malay, 72-year old

“… all my children support me to do gardening as they knew that was my passion. They will show me pictures of ornamental plants from websites and will ask me whether I need one to plant in my house. They support me by buying fertilizers and flowerpots … they knew that I need my own time to relax and do things I like”

Malay, 67-year old

Many participants expounded that they felt being emotionally supported when they were encouraged by the doctors to engage with physical activities to stay active. In spite of the advice and guidance on how to be physically active, expression of emotional support through the care and encouragement to continuously partake in physical activities was another form of a primary motivating factor for the older women to stay active in their daily lives. The participants also registered that they were emotionally inclined to the doctors when they were promoting physical activities to create awareness of the healthy benefits of being physically active.

“The doctor from Klinik Kesihatan Kuala Pilah (Kuala Pilah Clinic) recommended me to exercise often other than taking my daily medication. I felt grateful for his concern and encouragement to be active when the doctor was taking extra care of my health. So, I need to do my part …”

Malay, 68-year old

“The doctor said that it was normal to experience bodily aches at this age. He said that we still need to be active irrespective of the bodily pains to stay healthy… The doctor is more concerned about our health compared to us (laughing)”

Malay, 60-year old

#### 3.2.2. The Absence of Social Support as a Motivator to Engage the Older Women with Physical Activities

The findings of this study also surfaced the theme of the absence of social support as a motivator to engage with physical activities among older women. For instance, some of the participants clarified that they were engaging in physical activities due to the absence of someone who used to help them when the person was alive or physically present. They indicated that the loss of a person that they perceived as a source of hope and help had changed their behaviour to be more independent and active to accomplish all their tasks on their own. The findings indicated that the older women experiencing a greater loss for social support from the loved one and managing it by becoming more independent in their lives. For instance, one of the participants registered that the demise of her daughter who used to help her had forced her to perform all the daily activities by herself.

“I had a daughter last time. She helped and cooked for me. Being an old lady, she was a great source of hope and help for me. Now, she is no more and I have to do all my tasks myself”

Malay, 71-year old

The findings revealed that some older women were willing to perform their tasks, housework and other activities such as buying groceries and paying bills as they were unwilling to burden their family members who were literally staying apart from them. In fact, some older women registered that they were more receptive to obtain help and support from their friends and peers since they perceived the emotional and instrumental qualities of the friendship to be less burdening over family members. Therefore, they were willing to engage with their friends to accomplish their work rather than waiting for their family members to come and help them.

“After I retired, I enjoyed travelling. I want to keep myself active. I am alone, I want to keep myself independent and I do not want to trouble my children, especially when they are working and living far from me. I keep myself mobile, active and don’t want to burden them”

Chinese, 73-year old

“I usually cook my food myself as I don’t want to burden my children. I will feel sorry if my children have to cook for me or clean my bedroom after they come back from their work. They are tired too …”

Malay, 82-year old

#### 3.2.3. Lack of Social Support and Less Supporting Social Environment as Barriers of Physical Activities among Older Women

The theme of lacking social support as a barrier to physical activities emerged as a component that negatively influenced physical activity behaviour among older women. The findings of the study indicated that older women’s participation in physically-oriented leisure activities such as walking and jogging were likely affected when they lost the companionship and appraisal support from their friends. The lacking absolute companionship and instrumental assistance by their friends in terms of co-participation in leisure activities and emotional support in terms of giving encouragement and offering positive feedback were reported as posing an incremental effect on physical activity behaviour among the older women in rural areas.

“I felt like I’ve lost strong support after the death of my close friend. She will accompany me wherever I go. Now, I’m no more going out as I have no companionship of hers”

Malay, 72-year old

“I’m not scared to walk outside. However, I have no friends to walk along with. I’m willing to walk if my friends were around to walk with me”

Malay, 86-year old

Some participants described lacking support and encouragement from their children as one of the demotivating factors to be active in their lives. Some explained that their children were making fun of their age, physical appearance and ability to participate in outdoor aerobic exercises. This became one of the factors for some participants to not preferring group activities and exercises that would be made them feel embarrassed by their age and appearance. Some were discouraged by their children to be less fit to the competitive atmosphere of group activities or apprehensive about not being able to keep a comfortable pace in the group activities.

“My children used to make fun out of me that I still wanted to go for aerobic exercises at this age. They said that I’m old and could find it difficult to participate in exercise sessions along with the other participants”

Malay, 72-year old

Despite friends and children being the source of social support, the older women with a reduced circle of friends and children staying far from them may find that the spouses were the only source of social relations that support them to engage in physical activities. Nevertheless, lacking spousal support to encourage, motivate and drive them to attend exercise sessions and group activities will eventually make them be less active. For example, some participants complained that spouses refused to drive them to the nearest exercise classes and programmes potentially due to other commitments, which further hindered them from engaging with physical activities.

“I’m aware of the existence of aerobic exercise programmes at Klinik Kesihatan Kuala Pilah (Kuala Pilah Clinic). However, my husband has no time to send me to the clinic to attend the programme. I don’t want to force my husband too”

Malay, 67-year old

It was eminent that poor safety and security issues in the surrounding neighbourhood were one of the many barriers that emerged as barriers of physical activities among older women in this study. Findings suggested that poor neighbourhood safety reduces the number of outdoor activities for older women. In particular, perceptions of poor neighbourhood safety may reduce the participation of older women in physical activities out of their fear of neighbourhood crimes. One of the participants informed that:

“I felt scared to walk outside of my house as my neighbourhood is not safe enough for outdoor activities. Previously, some people disguised as government officers and attempted to cheat the locals here”

Malay, 68-year old

Some participants described the logistic issues and unavailability of public transportation to bring them to the nearest exercise centres or to attending exercise programmes as one of the factors that prevented them from engaging in physical activities.

“I can’t attend the aerobic exercises for older people that they organize in the clinics as I don’t have my own transportation to go there”

Malay, 71-year old

“My kids are working. Even if I’m willing to attend the exercise programmes for the older people, I still can’t attend as there is no public transportation available near my residence to bring me there”

Malay, 68-year old

## 4. Discussion

This study presented a qualitative analysis and visualization on the role of social support towards physical activity behaviour among rural community-dwelling older women in Kuala Pilah District, Negeri Sembilan. The overall findings underlined mixed responses on the roles of social support on physical activity behaviour among the older women that gave rise to three categories of themes. Ultimately, adaptive social support in terms of informational, companionship and emotional supports has encouraged engagement in physical activities among older women. The absence of social support was reported to make older women more independent and trigger them to engage in physical activities. On the contrary, there were also reports on the lack of social support which demotivated participation in physical activities among older women. These findings implied that whilst adaptive social support can greatly increase physical activity levels among older women, poor social support could render both positive and negative influences on physical activity engagement. These findings were in line with Rook et al. [41] who described that the social network members may use positive social control strategies that encourage more activity in a constructive manner. Similarly, they may use negative strategies such as pressures or criticism of inactivity to make the older women be physically active.

The main findings suggested that the availability of informational support such as advice, guidance, suggestions and other useful information from friends, media and healthcare practitioners could greatly influence the physical activity behaviour via perceived behavioural control [42,43]. The information received from the social network and surrounding environment anticipated promoting self-confidence and positive thoughts that boost their energy to be physically active than those who lack the self-confidence [44]. The attitude of a social network member towards a particular physical activity could influence the other members towards a changed behaviour [45,46]. For instance, an invitation to joint activities such as jogging, walking and aerobic exercises by the friends were envisaged as a form of persuasion to engage with physical activities among the other older women. Despite being a remedy to old age loneliness, the attitude and fear of letting the friend down on the occasion of stopping group exercises seen as a motivation factor to be continuously engaged with physical activities. Peer companionship could additionally provide them with a sense of social belonging while undertaking physical activities with their peers [43].

This study outlined the information and exposure obtained from the media targeting older people, including religious views on physical activities, as a form of a motivator for older women to engage with physical activities. In the local context, older women’s traditional and religious belief that by being a woman one needs to serve the family and children demonstrated their ideology on gender-specific responsibilities to be active in the routine lifestyle [47,48]. In another aspect, the roles of media must be strengthened to be an effective communication tool to occasionally convey the messages and information related to the health risks due to inactivity that engage the older women in physical activity. The efficiency of social media such as Facebook and YouTube channels also need to be improved as an efficacious informational tool in providing examples of exercises and physical activities suitable for older women to engage with physical activities.

Additionally, findings from this study echoed other studies that demonstrated the role of effective patient-doctor communication and interaction in boosting physical activities by imparting valuable information related to the types, benefits and methods of performing physical activities among older adults [49]. In line with this, physical activity interventions based in primary care centres may be effective in older women as they may be more responsive to their healthcare professionals’ advice and guidance potentially because they value health advice from their doctors as professionals in the field. However, it is notable that although health programmes have been structured within primary health clinics, logistic issues and lacking supports from spouses and children could limit older women’s participation in physical activities. Hence, it is recommended that the health care system should implement educational sessions for spouses and children of older women to create more awareness of their roles and responsibilities towards the older women.

Emotional support in the form of concern and love from the social network including family and friends was perceived to motivate women to engage in physical activities. As such, emotional support may shape self-efficacy or optimism on the benefits of the physical activities, which in turn, support their decisions to be physically active [32,41,50]. However, the expression of emotional support can vary across members of social networks and can be perceived differently by older women. For example, emotional support from family members could be perceived as more important compared to other members of the network potentially due to the bond or connection between the family members [50]. In this study, we noted that emotional support was gained from the family members able to motivate the older women to be engaged with physical activities. Such scenario accentuated the need for the interventions to focus on creating a supportive atmosphere for physical activity both in the home and in social circles of older women in order to increase the chances of successful behaviour change [41,51,52].

Contrary to earlier reports in this study on the positive influence of social support on physical activities among older women, the absence of social support has also promoted resilience among older women. In particular, the results showed that the older women experience a greater loss from the loved one for social help and manage it by being more independent in their lives. Being self-reliant has led older women to perform household chores, especially when there was no help available from anyone in their social network [52,53]. While some participants described the absence of social support, there were also reports on the minimal involvement of members in the social network. Specifically, some older women were willing to perform their own tasks, housework and other activities as they were unwilling to burden their family members who were literally staying apart from them. Collectively, self-help strategies that were used to maintain independence in daily tasks such as performing household chores could keep the elderly people become active in addition to social connections, natural and built environments [54].

Poor social support has been demonstrated to have a negative influence on physical activity engagement. For instance, the lack of acceptance from children was perceived as one of the demotivating factors to be physically active among older women. Even though children’s belief in the capability of older women could act as the biggest motivating factor to amplify the physical activities and physical competence among them, such beliefs were not observed from the narratives [55,56,57]. Even though social supports such as persuasion can be treated as a pushing factor to encourage physical activities, it can produce unintended reverse actions, leading to a lack of engagement in physical activities [51,52]. As discussed by Hess and Davis [58], older women could consider that they were engaged with normal routine activities in the house and hence engaging in other forms of physical activities was unnecessary. Such thoughts can be amplified when they were not supported by their own spouses and children and tend to allocate lesser or no time for exercise. Therefore, spouses could encourage women to perform physical activities and emphasis on educational sessions targeting men to encourage their wives to exercise is equally important to foster physical activity behaviour among the older women. Additionally, older women perceived neighbourhood safety as a barrier to physical activities. The community, as part of their social support, should ensure that the neighbourhood is friendly and safe for the older women to perform physical activity. Improvement in the safety and security features of the neighbourhood is essential to increase the participation of older women in physical activities [59,60]. Being qualitative in nature, this study has several limitations that need to be considered when interpreting its findings. The participants were interviewed in their native language before being translated into English. There could be some potential translation errors and the nuances could have lost during translations. Social desirability is another limitation where some participants tend to provide their views in a way they deem to be more socially acceptable to avoid receiving negative evaluations. The study’s participants are mostly Malay women, and therefore the inputs from women of other ethnic groups could be underestimated. In spite of these methodological caveats, this study provides useful insights on the influence of social support on physical activity behaviour to devise evidence-based interventions as discussed above targeting physical activity improvements among rural community-dwelling older women.

## 5. Conclusions

In conclusion, the overall findings underlined that whilst adaptive social support can enhance physical activity levels among older women, poor social support could render both positive and negative influences on their physical activity levels. The findings reported in this study has strong public health implications to tailor interventions, necessary actions and educational campaigns among the older women and members of their social network to emphasize their roles in encouraging the older women to be physically active. Religious programmes emphasizing the value of maintaining an active lifestyle, broadcasted on the television and radio channels, may be a particularly potent means of motivating older women to be physically active and educating families about the importance of emotional and companionship support, especially from the spouses and children, may also potentially create an enabling environment for older women in rural settings. In addition, the roles of social media and effective patient-doctor communication must be strengthened to create a supportive atmosphere for older women to do physical activities.

## Figures and Tables

**Table 1:** Semi-structured interview guide and probes.

Themes	Issues	Probes
Types of social support	What types of support do you receive from your social network to engage in physical activities?	(1) How do you feel to engage in physical activities due to the social support that you are receiving from the people in your surroundings? (2) Why are you engaging in physical activities when there are people to take care of you or do you have no support or choices to be made (eg: being alone, no one to take care of, working, etc)? (3) Do your social network provide sufficient love, care, trust and acceptance to encourage you to indulge in physical activities? (4) What do you feel when your companions and part-ners invited you to carry out shared activities? (5) What do you feel with the presence of companions while engaging in shared activities? (6) How your family, friends and neighbours assist and help you when you wanted to engage in any physical activities both indoor and outdoor? (7) Do your family, friends and neighbours motivate, advise and guide you to engage in physical activities?
The importance of social support	Do you think that the social support received from the family, friends and neighbours is important for you to be active?	(1) How do you feel when receiving social support from the family, friends and neighbours to be active? (2) What do you think might happen if there is no social support given by the family, friends and neighbours to you?

**Table 2:** Socio-demographic attributes of the participants.

Socio-Demographic Characteristics	Frequency (*n*)	Percentage (%)
Age (years)		
Mean age (SD)	74 ± 5.9 years	NA
Range	60–90 years	NA
Ethnicity		
Malay	13	76.5
Chinese	2	11.8
Indian	2	11.8
Others	0	0
Religion		
Muslim	13	76.5
Buddhism	2	11.8
Hinduism	2	11.8
Christianity	0	0
Others	0	0
Educational level		
Primary education	5	29.4
Secondary education	12	71
Tertiary education	0	0
Marital status		
Single	0	0
Married	17	100
Occupation		
Professional and managerial	0	0
Skilled worker	0	0
Housewife	14	82.4
Retired or unemployed	3	17.6

## Data Availability

The datasets generated and analysed during the current study can be obtained from the first author or corresponding author on reasonable request.
